# Whole genome sequence analysis of Australian avian pathogenic *Escherichia coli* that carry the class 1 integrase gene

**DOI:** 10.1099/mgen.0.000250

**Published:** 2019-01-23

**Authors:** Max L. Cummins, Cameron J. Reid, Piklu Roy Chowdhury, Rhys N. Bushell, Nicolas Esbert, Kelly A. Tivendale, Amir H. Noormohammadi, Shaiful Islam, Marc S. Marenda, Glenn F. Browning, Philip F. Markham, Steven P. Djordjevic

**Affiliations:** ^1^​ The ithree Institute, University of Technology Sydney, Ultimo, NSW, Australia; ^2^​ Asia-Pacific Centre for Animal Health, Department of Veterinary Biosciences, Faculty of Veterinary and Agricultural Sciences, The University of Melbourne, Parkville, Victoria 3010, and Werribee, Victoria 3030, Australia

**Keywords:** microbial genomics, genomic epidemiology, *Escherichia coli*, avian pathogenic *E. coli*, antimicrobial resistance, whole genome sequencing

## Abstract

Avian pathogenic *Escherichia coli* (APEC) cause widespread economic losses in poultry production and are potential zoonotic pathogens. Genome sequences of 95 APEC from commercial poultry operations in four Australian states that carried the class 1 integrase gene *intI1*, a proxy for multiple drug resistance (MDR), were characterized. Sequence types ST117 (22/95), ST350 (10/95), ST429 and ST57 (each 9/95), ST95 (8/95) and ST973 (7/95) dominated, while 24 STs were represented by one or two strains. FII and FIB *repA* genes were the predominant (each 93/95, 98 %) plasmid incompatibility groups identified, but those of B/O/K/Z (25/95, 26 %) and I1 (24/95, 25 %) were also identified frequently. Virulence-associated genes (VAGs) carried by ColV and ColBM virulence plasmids, including those encoding protectins [*iss* (91/95, 96 %), *ompT* (91/95, 96 %) and *traT* (90/95, 95 %)], iron-acquisition systems [*sitA* (88/95, 93 %), *etsA* (87/95, 92 %), *iroN* (84/95, 89 %) and *iucD*/*iutA* (84/95, 89 %)] and the putative avian haemolysin *hylF* (91/95, 96 %), featured prominently. Notably, mobile resistance genes conferring resistance to fluoroquinolones, colistin, extended-spectrum β-lactams and carbapenems were not detected in the genomes of these 95 APEC but carriage of the sulphonamide resistance gene, *sul1* (59/95, 63 %), the trimethoprim resistance gene cassettes *dfrA5* (48/95, 50 %) and *dfrA1* (25/95, 27 %), the tetracycline resistance determinant *tet(A)* (51/95, 55 %) and the ampicillin resistance genes *bla_TEM-1A/B/C_* (48/95, 52 %) was common. IS*26* (77/95, 81 %), an insertion element known to capture and mobilize a wide spectrum of antimicrobial resistance genes, was also frequently identified. These studies provide a baseline snapshot of drug-resistant APEC in Australia and their role in the carriage of ColV-like virulence plasmids.

## Data Summary

1. Ninety-five pairs of short-read data of avian pathogenic *E. coli* sequenced for this study have been deposited in the NCBI Short Read Archive under Study ID 479542. Additionally, draft genome assemblies have also been uploaded and are accessible under this same study ID. Individual sample accession numbers can be found in Table S1 (available at the online version of this article).

https://www.ncbi.nlm.nih.gov/bioproject/PRJNA479542

**Impact Statement**

Avian pathogenic *Escherichia coli* (APEC) are known to carry an impressive arsenal of virulence-associated genes (VAGs), several of which are known to facilitate invasion of epithelial cells and survival in poultry, presumably enabling APEC to disseminate from their initial site of colonization in the respiratory tract to multiple organ sites. This is the first study that describes the genetic composition of drug-resistant APEC in Australia. It indicates that Australian APEC belong to sequence types (STs) that carry a diverse array of VAGs, many of which are highly related to extraintestinal pathogenic *E. coli* recovered from human patients with a variety of associated diseases. VAGs encoding iron acquisition systems, toxins and factors that promote survival in human urine and blood co-residing on ColV-like IncF virulence plasmids were identified in our study. Notably, we identified high carriage rates of IS*26*, an insertion element that is thought to play an important role in the evolution of antimicrobial resistance regions. Further studies are needed to determine the role played by IS*26* in the assembly of complex resistance regions on ColV-like and other APEC plasmids.

## Introduction

*Escherichia coli* are considered the most frequently isolated Gram-negative pathogen affecting human health [1]. Over the past 20 years, extraintestinal pathogenic *E. coli* (ExPEC) have risen to prominence. ExPEC colonize the gut asymptomatically, but carry virulence-associated genes (VAGs) that enable them to colonize extraintestinal sites and cause disease. Most ExPEC infections localize to the urinary tract and are known as uropathogenic *E. coli* (UPEC). UPEC can progress from the bladder to cause more serious disease, including pyelonephritis and sepsis (uroseptic *E. coli*). Another subset of ExPEC, neonatal meningitis-causing *E. coli* (NMEC), can produce severe neurological disease, particularly in infants. ExPEC also cause disease in diverse, agriculturally important, food animal species, particularly poultry (avian pathogenic *E. coli*; APEC), but also in swine and dairy cattle [2–5]. Infections caused by multiple drug-resistant (MDR) ExPEC are increasing in frequency and are a major cause for concern [6].

APEC carry large conjugative plasmids containing combinations of iron acquisition genes, including the *iucABCDiutA* (aerobactin uptake) and *iroBCDEN* (salmochelin uptake) operons, as well as other heavy metal transporters *sitABCD* and *etsABC*, and the serum resistance gene *iss*. The carriage of these VAGs has been linked to the capacity of APEC to cause disease, but their presence is not essential for extraintestinal infection in an avian host [7–12]. APEC are genetically heterogenous and carry diverse combinations of VAGs involved in iron acquisition, cytotoxicity, adhesion, invasion and immune evasion. Many of the putative virulence genes found in APEC are also found in human ExPEC. APEC can grow in human urine, resist mammalian complement and invade human epithelial cells [13–16]. Moreover, APEC and human ExPEC share serotypes, sequence types (STs) and PFGE profiles [17–19]. Collectively, these and other observations underpin the hypothesis that poultry-associated *E. coli* pose a zoonotic threat [4, 5, 15, 20–22], although the zoonotic potential of APEC has yet to be quantified [23, 24]. While animal models seeking to determine the zoonotic potential of APEC have been informative, they have not been definitive. Reverse zoonotic episodes where MDR pathogens carried by humans are transferred to poultry and other food animals also pose a significant biosecurity threat [25].

APEC are found in the intestinal flora of healthy commercial bird species, can cause disease at various anatomical sites, and are a leading cause of mortality and morbidity in poultry of all ages [3, 26]. APEC infect the trachea and air sacs, as well as the oviduct, pleura, peritoneum and pericardium, liver, blood, yolk sac, growth plates and joints [27]. Many systemic infections caused by APEC are initiated by colonization of the respiratory tract after inhalation of faecally contaminated dust, with subsequent dissemination to more distant sites. However, the factors that precipitate invasion are not well understood [21, 27–30].

The introduction of APEC plasmids into avian commensal *E. coli* has been shown to confer virulence in animal models of ExPEC disease [31]. APEC are often resistant to a range of antimicrobial agents, including tetracyclines, chloramphenicol, sulphonamides, aminoglycosides, fluoroquinolones and β-lactams [32] and the corresponding resistance genes are often plasmid-associated. The introduction of MDR plasmids from poultry via food into the human gut is a potential threat to human health. Whole genome sequencing (WGS) approaches will help to provide insights into the zoonotic potential of APEC and into the role of mobile DNA in pathogen evolution and assembly and the spread of antimicrobial resistance genes [33, 34], although WGS studies of APEC are in their infancy [35], particularly within Australia.

Here, we used WGS to characterize 95 geographically diverse APEC strains that had been determined by PCR to carry a class 1 integrase (*intI1*) gene, a reliable proxy for MDR [36], which here is defined as carriage of three or more genes associated with resistance to different classes of antibiotic. The genome sequences were interrogated for the Clermont phylogroup, e-serotype, multi-locus sequence type (MLST) and VAGs, to seek novel insights into the genetic characteristics of Australian APEC carrying multiple antimicrobial resistance genes.

## Methods

### Sample origins and associated metadata

The APEC that were investigated had diverse origins. They were obtained between 2007 and 2015 from at least 12 Australian agricultural poultry operations across four states (Victoria and New South Wales, Queensland and Western Australia), although as the geographical origins of some isolates were unclear, the exact number of sources cannot be determined. The APEC originated predominantly from broiler and layer chickens, and to a lesser extent from turkeys and ducks (File S1). Data on any antimicrobial therapy administered to these animals are limited, but in Australia relatively few antimicrobials are approved for commercial poultry and the administration of several active ingredients (e.g. gentamicin, fluoroquinolones and chloramphenicol) is not permitted and therefore extremely unlikely to have been used in the flocks. Samples identified to have originated from the same geographical site at the same time and that shared identical phylogenetic classifications and genotypes were considered duplicate isolates and removed from the analysis.

### Isolate collection

Swabs were collected from multiple anatomical sites by a team of experienced veterinarians from the University of Melbourne from deceased or culled birds with signs of an APEC infection. Anatomical sampling sites varied between birds, but in most cases samples were taken from internal organs. *E. coli* were cultured on sheep blood and MacConkey agar and a routine PCR [37] was used to determine whether the *E. coli* carried the typical repertoire of APEC VAGs. The isolates were stored at −80 °C in 20 % glycerol or on Protect (Thermo-Fisher) beads.

### Determination of *intI1* carriage by PCR

Single APEC colonies were picked from LB agar plates and inoculated in 5 ml of LB medium to prepare glycerol stocks and crude DNA templates for PCR [38]. Primers HS915/HS916, which span a 371 bp region of *intI1*, were used to identify isolates carrying a class 1 integron, as previously described [39]. Isolates that yielded a band of 371 bp amplicon indicative of the *intI1* gene were selected for WGS.

### DNA extraction, WGS and assembly

Genomic DNA was extracted using the ISOLATE II Genomic DNA Kit (Bioline) following the manufacturer’s instructions for bacterial cells and stored at −20 °C. Library preparation was undertaken using Nextera DNA Library Preparation kits generating 150 bp paired end reads from 0.5 ng of template DNA. WGS of strains was performed using an Illumina HiSeq 2500. Sequence read quality was assessed using FastQC version 0.11.5 (http://www.bioinformatics.babraham.ac.uk/projects/fastqc/) before Illumina raw reads passing quality control were assembled into draft genome sequences using the A5 assembly pipeline version A5-miseq 20150522 [40]. Genomes with an average read depth of ≥20, and that also assembled to 600 or fewer scaffolds, were retained for further phylogenetic analysis using Phylosift. Draft genome assemblies were deposited in NCBI; individual accession numbers can be found in Table S1.

### Genotyping and phylogenetic classification

Publicly available databases such as PlasmidFinder, ResFinder, VirulenceFinder (http://www.genomicepidemiology.org/) and ISfinder [41] were used to source reference sequences for genotyping, with additional sequences of interest not present within these databases collected from the NCBI nucleotide database and the Virulence Factor Database [42]. Genotyping, including for the purposes of phylogroup [43] and e-serotype classification [44], and MLST (http://mlst.warwick.ac.uk/mlst/) were performed using the read-mapping tool ARIBA [45] before the processing of such data with a bespoke script accessible on GitHub (https://github.com/maxlcummins/APEC-MGEN-2018).

### Single nucleotide polymorphism analyses

Phylogenetic SNP analysis and identification of SNPs in *gyrA* and *parC* conferring fluoroquinolone resistance (*gyrA*: Ser-83-Leu, Asp-87-Asn; *parC*: Ser-80-Ile, Glu-84-Gly) [46] was performed using Snippy version 3.2 [47], with a K12 strain used as a reference (accession no. KU00096.3), and manually curated through use of AliView [48].

### Phylogenetic analyses

Maximum-likelihood phylogenetic tree analyses were performed under a generalized time-reversible model using the PhyloSift pipeline version 1.0.1 [49] and FastTree version 2.1.8 [50], altered to resolve short branches as previously described [51], and visualized in iTOL [52].

Phylogenetic SNP trees were generated using Snippy, with the resulting full core genome alignment filtered for recombination with Gubbins (https://sanger-pathogens.github.io/gubbins/). SNP-sites v2.4.0 (https://github.com/sanger-pathogens/snp-sites) was then used to create an alignment consisting of 1539 variable sites before tree generation with FastTree [50], also using a generalized time-reversible model. This tree was visualized using the R package ggtree [53]. SNPs were counted using snpiphy (https://github.com/bogemad/snpiphy). Additional details are available at https://github.com/maxlcummins/APEC-MGEN-2018.

### Inference of ColV-like virulence plasmid carriage

Short reads from each sample were mapped to the reference plasmid pCERC4 (accession no. KU578032) using the Burrows–Wheeler Aligner (BWA) 0.7.17 [54] and converted to a BAM file format using SAMtools 0.1.18 [55]. Through use of a bespoke Python script each BAM file was then used to produce a histogram of read-depth as a function of reference coordinate, clustered based on their euclidean distances, and used to generate a heatmap. A schematic of pCERC4 was then generated using SnapGene (https://www.snapgene.com/) and overlayed above the heatmap to facilitate visualization of the relative genetic loci where sample reads were mapped. The scripts and commands used are accessible on GitHub (https://github.com/maxlcummins/APEC-MGEN-2018).

## Results and Discussion

### ST117 and ST350 are predominant lineages

**Fig. 1.**
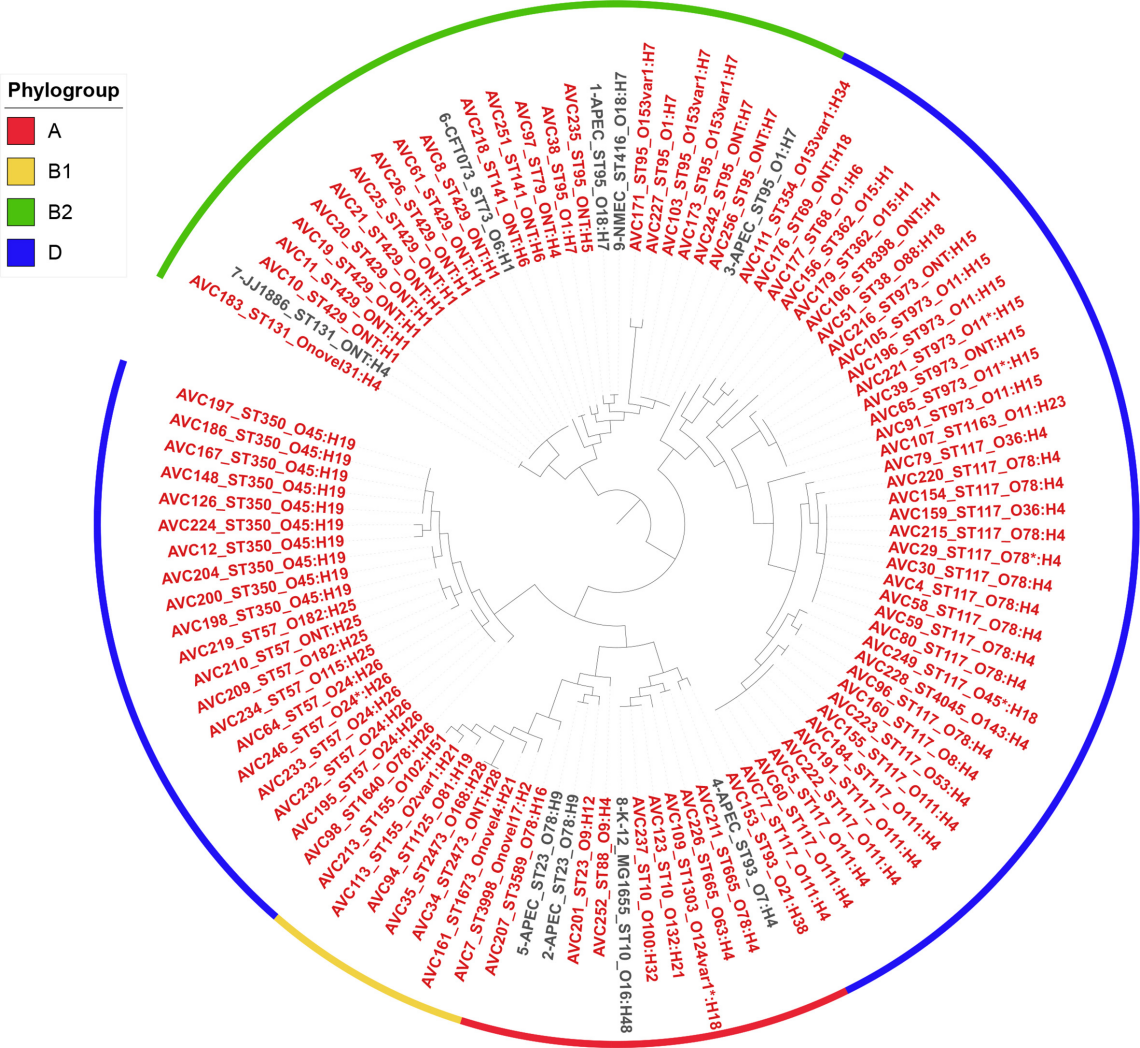
Phylogenetic relatedness of the APEC isolates and nine reference strains (accession numbers: 1, CP006830; 2, CP007442; 3, NC_008563; 4, CP013048; 5, CP004009; 6, AE014075; 7, CP006784; 8, U00096; 9, CP007275), as determined by PhyloSift in combination with FastTree. The tree is midpoint rooted. Red tip labels indicate isolates from the present study, while those shown in black are the reference strains. The STs and e-serotypes of isolates have been appended to their names (e.g. A5_ST117_O111:H4). The coloured bands encircling sections of the tree indicate the phylogroups into which isolates were categorized. Asterisks in sample names indicate low read depth at one or more loci for a given MLST/O-type/H-type (see https://www.github.com/maxlcummins/ARIBAlord for more information).

Out of 256 APEC isolates, 123 were predicted by PCR to carry a class 1 integron and were sequenced. Twenty-six isolates failed to be sequenced and/or assembled to a quality that met our aforementioned assembly criteria, while an additional two isolates were considered duplicates and removed from further analysis. Assembly statistics of samples analysed are shown in Table S1. Based on comparisons of 37 core protein sequences from PhyloSift, these remaining 95 APEC clustered into five clades and were heterogeneous in nature (Fig. 1). Genomes sharing the same ST and e-serotype clustered, as expected. Analysis revealed that phylogroup D (58/95) and, to a lesser extent, B2 (21/95) were dominant in Australian *intI1*-positive APEC populations, cumulatively representing 82 % (79/95) of the collection. The distribution of APEC between phylogroups appears to vary in different countries. While APEC within phylogroups B2 and D [35, 56] usually predominate, some APEC populations can also contain high proportions of members of phylogroup A [57]. The increased frequency of typically commensal phylogroups A and B1 among ExPEC and APEC [35] suggests that pathogenic *E. coli* continue to evolve rapidly via mechanisms underpinned by lateral gene transfer.

Thirty STs were identified among the 95 APEC. There were several dominant lineages, including ST117, clonal complex 350 (CC350) (ST350, ST57), ST95, ST429 and ST973, which cumulatively represented 64 of the 95 APEC. Notably, ST117 was the most frequent ST (22/95) in the collection. ST117 is a major cause of extraintestinal infections in poultry, is considered an emerging human pathogen globally [58, 59], and is noted for its carriage of genes encoding extended-spectrum β-lactamases [60]. APEC-associated ST117 isolates are serotypically diverse, but most have H4 flagella [35, 61–63]. Among our 23 CC117 isolates (ST117, 22 isolates; ST4045, one isolate), seven serotypes were identified, but serotypes O78:H4 and O111:H4 predominated. These serotypes have also been identified in studies of APEC in Brazil and several Nordic countries [58, 63–65], suggesting that they may represent globally disseminated subclones.

**Fig. 2.**
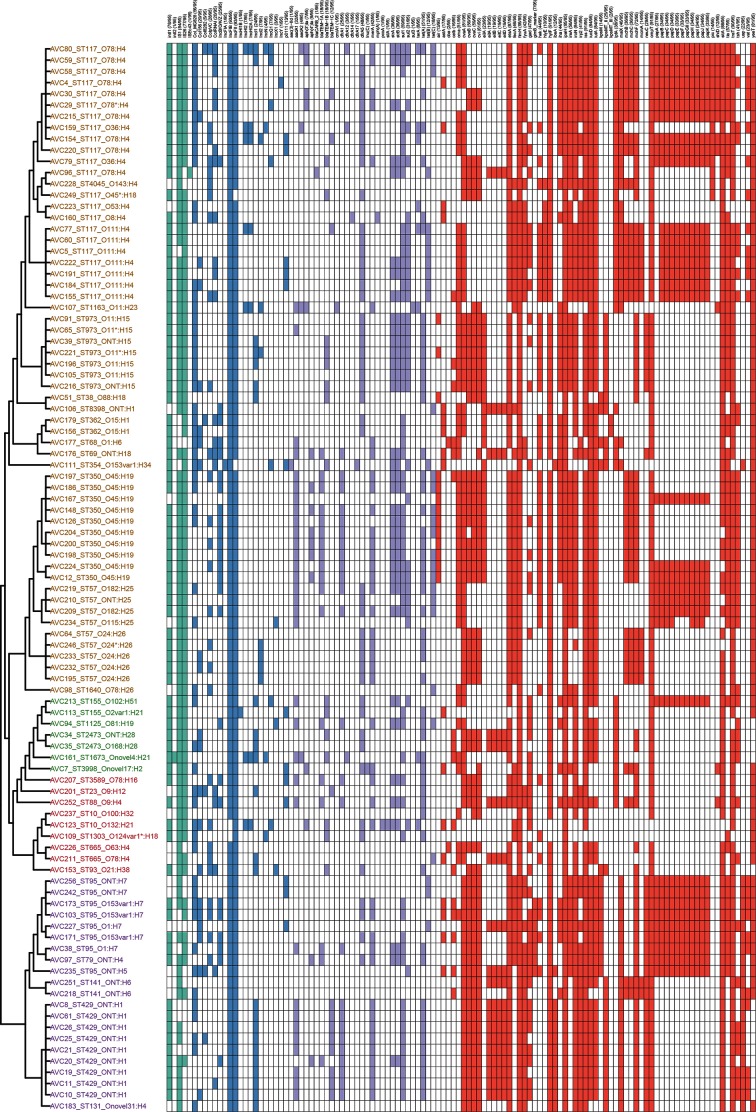
Genotypic profiles of APEC isolates, clustered on the basis of the PhyloSift tree in Fig. 1, with the tip labels indicating the ST and e-serotype (in text) and phylogroup (indicated by text colour, where A is red, B1 is green, B2 is purple and D is mustard). Carriage of mobile genetic element-associated genes (teal), AMR genes (purple), VAGs (red) and plasmid *repA* genes (blue) are shown adjacent to the tree in a hit-table, with a white square indicating the absence of a specific gene.

Although the genotypic profiles of the 23 CC117 isolates were variable, all were rich in VAG content, particularly those in serogroups O78 and O111 (Fig. 2). All CC117 isolates carried *etsA*, *iucD*/*iutA* and *sitA*, while *ireA* (22/23, 96 %), *iroN* (21/23, 91 %) and *fyuA* (20/23, 87 %) were also common. The protectin-associated genes *iss* and *ompT* were also ubiquitous in these isolates, and 91 % (21/23) also carried *traT*. Genes thought to be involved in host-cell adhesion were also common (*irp2* and *tia*, 9/23, 83 %; *papGII*, 17/23, 74 %). Plasmid replicon types within this CC were quite diverse, with *repA* genes associated with nine different incompatibility groups represented. The most common were FIB (23/23, 100 %), FII (22/23, 96 %) and HI2 (4/23, 17 %). IncN (2/23, 9 %), IncI1 (2/23, 9 %), IncI2 (1/23, 4 %) and IncY (1/23, 4 %) were also detected.

**Fig. 3.**
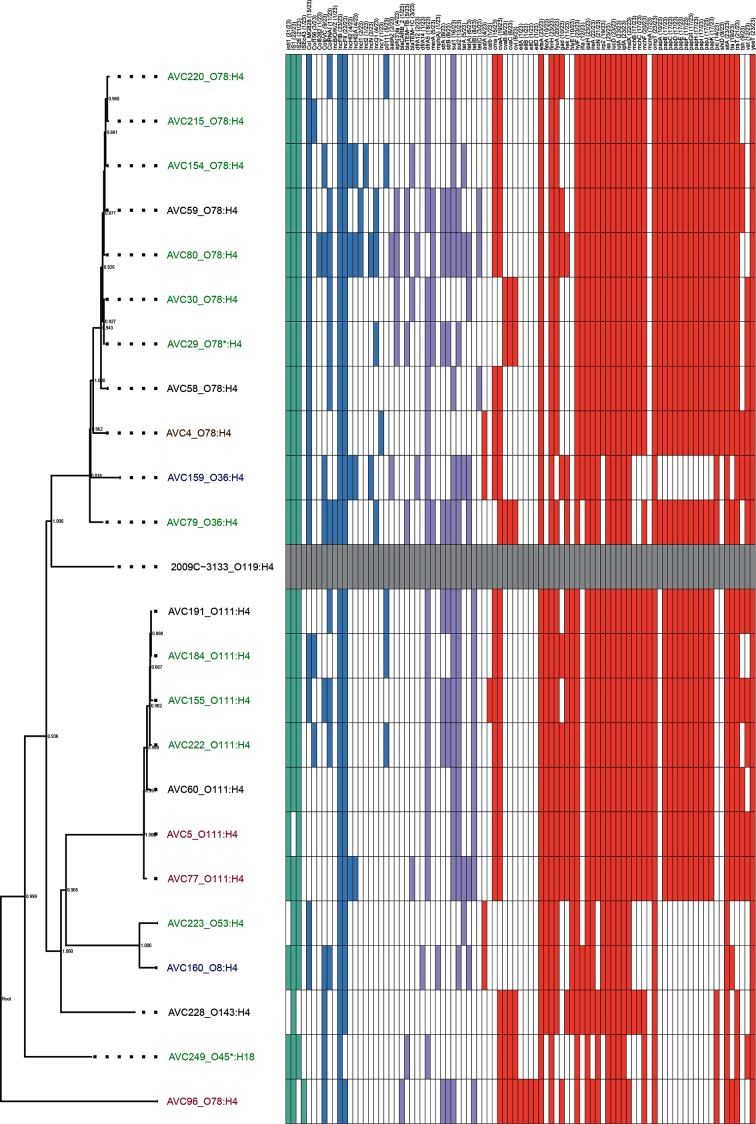
High-resolution phylogenetic comparison of ST117 APEC isolates, as determined by Snippy in combination with Gubbins, SNP-sites and FastTree. The tree is midpoint-rooted. The ST and e-serotype are shown on the tip labels, while the colour of the labels indicates the state of origin (brown, QLD; green, VIC; red, WA; blue, NSW; black, unknown). A gene hit map is also shown; mobile genetic element-associated genes (teal), AMR genes (purple), VAGs (red) and plasmid *repA* genes (blue) are shown adjacent to the tree in a hit-table, with a white square indicating the absence of a specific gene.

The mean and median SNP counts across all CC117 isolates, relative to 2009–3133, were 277 and 348, respectively, with 57 % (13/23) of the isolates having 50 or fewer SNPs when compared to one or more CC117 isolates within the collection. While some of the CC117 isolates of the same serotype exhibited low SNP counts across their core genome, there were also examples of serotypically homogenous isolates that had high SNP counts (Fig. 3; Table S2). For example, isolates AVC77 and AVC222 were both serotype O111:H4 but differed from each other by only 23 SNPs, even though AVC77 was isolated from a bird from Western Australia in 2008 and AVC222 was isolated from a bird from Victoria in 2012; these are states separated by more than 1000 km. In contrast, two other isolates with the same serotype, O78:H4 (AVC96 and AVC29), were collected from these same states just one year apart and yet differed by 497 SNPs.

Apart from the 23 isolates of CC117, other prevalent lineages were ST57 (9/95; 9.5 %) and ST350 (10/95; 10.5 %), which belong to the same CC (CC350). The 19 members of this CC all carried *ompT*, *iucD*/*iutA*, *iss*, *iroN* and *hylF*. Other VAGs identified within this population included the *pap* operon, found in 37 % (7/19) of samples, and the adhesin *tsh*, which was found in 79 % (14/19) of isolates. APEC belonging to CC350 are frequently isolated from poultry in Australia and in other countries [56, 66, 67], are associated with extraintestinal infections including urinary tract infections (UTIs) and sepsis [67, 68], and may constitute a potential poultry-associated zoonotic agent. Two ST57 isolates from the collection under investigation were each found to carry a total of nine AMR genes, which, in combination with their extensive VAG profiles, highlighted them as a potential emerging pathogen.

Our APEC collection included 8/95 isolates belonging to ST95 and there were three different serotype profiles among them. These isolates had the highest level of carriage of extraintestinal VAGs; all carried *iroN*, *iss*, *kpsMT*(*II*), *iucD*, *ompT*, *papGII*, *neuC* and *usp*, while the gene encoding the vacuolated autotransporter toxin Vat and *gimB*, a marker of the genetic island linked to neonatal meningitis *E. coli* [21] and a capacity to invade cells [69], were carried by 6/8 of these isolates. ST95 is well documented in the literature as an APEC lineage that also frequently appears as a causative agent of UTIs and blood sepsis in humans [56, 70, 71].

The antimicrobial resistance (AMR) gene profiles of the eight ST95 isolates were variable, with one isolate carrying six AMR genes and the remaining isolates carrying only one or two. The ST95 lineage is unusual in that isolates are reported to have a lower level of acquired resistance than other pandemic lineages [71]. Fully assembled genomes of MDR ST95 contain resistance genes linked to large IncFIB/IncFII plasmids [72]. Plasmids with IncFII and IncFIB *repA* genes predominated in our collection and all eight ST95 isolates carried these markers. These data suggest that ST95 APEC of Australian origin, despite being variable in their AMR profiles, carry a significant reservoir of VAGs associated with human ExPEC infections, including UTIs, septicaemia and neonatal meningitis, and may have zoonotic potential.

### *intI1*-positive Australian APEC do not carry antimicrobial resistance genes of major clinical significance

We purposely targeted APEC that carried a class 1 integrase for WGS to maximize the likelihood of characterizing MDR strains. The most frequently identified AMR genes in the collection were *sul1* (59/95, 62 %), *tet(A)* (51/95, 54 %), *bla_TEM-1A/B/C_* (48/95, 51 %), *dfrA5* (48/95, 51 %), *strAB* (36/95, 38 %), *aadA1* (32/95, 34 %), *sul2* (31/95, 33 %), *dfrA1* (25/95, 26 %), *tet(C)* (18/95, 19 %), *tet(B)* (12/95, 13 %) and *aadA2* (4/95, 4 %) (Fig. 2).

The AMR genotypic profiles of these APEC isolates reflect the antimicrobial stewardship practices used widely in Australian poultry production systems. The most common phenotypic antimicrobial resistances reported in Australian APEC are to tetracycline, trimethoprim/sulfamethoxazole, streptomycin and ampicillin, at prevalences of 75, 38, 22 and 9%, respectively [73]. Our findings are largely consistent with these phenotypic resistance data, although we would have expected higher rates of resistance to ampicillin in the literature given the carriage rates of *bla_TEM-1_* in the APEC sequenced here.

Only one isolate, AVC111-ST354-ONT:H34, was found to have SNPs in *gyrA*/*parC* associated with fluoroquinolone resistance (FQR). This sample is of an ST *which* was reported in a study on FQR *E. coli* from canine faeces and cases of human ExPEC infection as a dominant lineage between both such sources [74], and therefore these *gyrA*/*parC* mutations are probably clonal. Additionally, analysis of APEC and Avian faecal *E.* *coli* (AFEC) in Australia identified strains of ST354 with FQR [75]. Otherwise, the APEC in our collection did not carry genes conferring resistance to antimicrobials important in the treatment of human disease, including cephalosporins, fluoroquinolones, carbapenems and colistin, an observation in stark contrast to those made on APEC isolated in many other countries. Studies in China and Egypt have reported that 75 and 23 % of APEC carry *bla*_CTX-M_ genes and *bla*_SHV_ genes [76, 77], respectively, while several studies on APEC from South Africa, China, Egypt and Vietnam have also identified APEC isolates carrying *mcr-1* [78–81].

We also failed to find any evidence of the carriage of genes encoding resistance to cephalosporins, fluoroquinolones, carbapenems or colistin among the genome sequences of porcine commensal *E. coli* that carry class 1 integrons [38]. This highlights the benefits of enforcing legislation to control use of critically important antimicrobials in food animals, as colistin, gentamicin, fluoroquinolones and amphenicol antimicrobials are not registered for use in food production animals in Australia (although restricted use of cephalosporins is allowed [82]). On a cautionary note, we detected IS*26* at a high prevalence in our APEC collection. IS*26* is an insertion element that forms composite transposons carrying a wide variety of antimicrobial resistance genes [83, 84], promotes cointegrated plasmid formation [85] and enhances plasmid fitness [86]. IS*26* can also recognize existing copies of IS*26* [87, 88] and promote formation of complex resistance gene regions [89, 90]. Therefore, long read sequencing would be useful in the investigation of the genetic context of the AMR genes detected, and other regions that abut insertion sequences such as IS*26*.

### Carriage of virulence-associated genes in APEC

Carriage of VAGs among Australian *intI1*-positive APEC isolates is shown in Fig. 2. Genes encoding iron capture systems were frequently represented. Specifically, *iutA* and *iucD* (aerobactin operon), *iroN* (salmochelin operon) and *sitA* (Sit operon) were often detected (84/95, 88 %; 84/95, 88 %; 84/95, 88 %; and 88/95, 93 %, respectively), while carriage of *ireA*, *irp2* and *fyuA* (Yersiniabactin operon) was less common (56/95, 59 %; 41/95, 43 %; 42/95, 44 %, respectively). The prevalence of these VAGs in Australian APEC is similar to that seen in APEC from other countries [37, 91–93]. Iron is tightly held in mammalian tissues and is a major factor limiting the growth of pathogens. APEC have evolved complex strategies, including the expression of specialized siderophores and iron chaperones, to recover iron from their host [26].

VAGs mediating protection against complement resistance are thought to be essential for the ability of APEC to disseminate to extrapulmonary sites. Almost all 95 APEC carried the increased serum survival gene *iss* (91/95, 96 %), *ompT* (91/95, 96 %) and *traT* (90/95, 95 %), genes that have been epidemiologically associated with or determined experimentally to confer serum resistance in ExPEC [94–97]. A recent study on 50 Australian APEC reported identical carriage rates of *iss* and *ompT* [75]. The importance of the *iss* gene as a marker of APEC is reinforced by its inclusion in a diagnostic pentaplex PCR [37]. Moreover, the Iss protein has been trialled as a heterotypically protective antigen in an experimental APEC vaccine [98]. More than a third (36/95) of the APEC isolates carried a variant of *kpsM*, the product of which is known to mediate complement resistance, a key characteristic of APEC. Group II variants of *kpsMT* are frequently detected among human ExPEC, but are less commonly detected in APEC globally. This locus was detected only within APEC in phylogroups B2 and D, which are historically associated with extraintestinal disease, with the notable exception of one ST93 isolate within phylogroup B1.

Many APEC-associated adhesins have been described, but their presence is not exclusive to APEC, so adhesin genes are poor diagnostic markers for APEC [12, 19]. All APEC in this study carried the fimbrial adhesin gene *fimH*, while 43 % (41/95) carried the putative adhesin *tsh* and 34 % (33/95) of the APEC isolates in this study carried *papGII* (pyelonephritis-associated pilus tip adhesin gene). Pap is thought to play a role in systemic extraintestinal infections of poultry and colonization of the kidneys in humans and the reproductive tract of dogs [99–103].

### Australian APEC frequently carry IncFIB and IncFII

Carriage of at least one plasmid *repA* gene was common in the Australian APEC studied here (Fig. 2). The most common *repA* genes belonged to Inc-types FII and FIB, which were each present in 98 % (93/95) of our APEC collection. Inc FII and FIB are commonly found in APEC globally [78] and are associated with large conjugative virulence plasmids that are a feature of the APEC phenotype [79]. IncB/O/K/Z and IncI1 incompatibility marker genes were also detected frequently (25/95, 26 %; 24/95, 25 %; respectively). An investigation by Johnson *et al*. in 2007 [_﻿_﻿^104^] found IncB/O/K/Z replicons at a similar prevalence (24 %) among a collection of 422 APEC and detected IncI1 at a slightly higher prevalence of 41 %. IncHI2, IncI2 and IncN *repA* genes were detected in 8 % (8/95), 7 % (7/95) and 4 % (4/95) of our Australian APEC isolates, respectively.

### Prevalance of ColV-like virulence plasmids

**Fig. 4.**
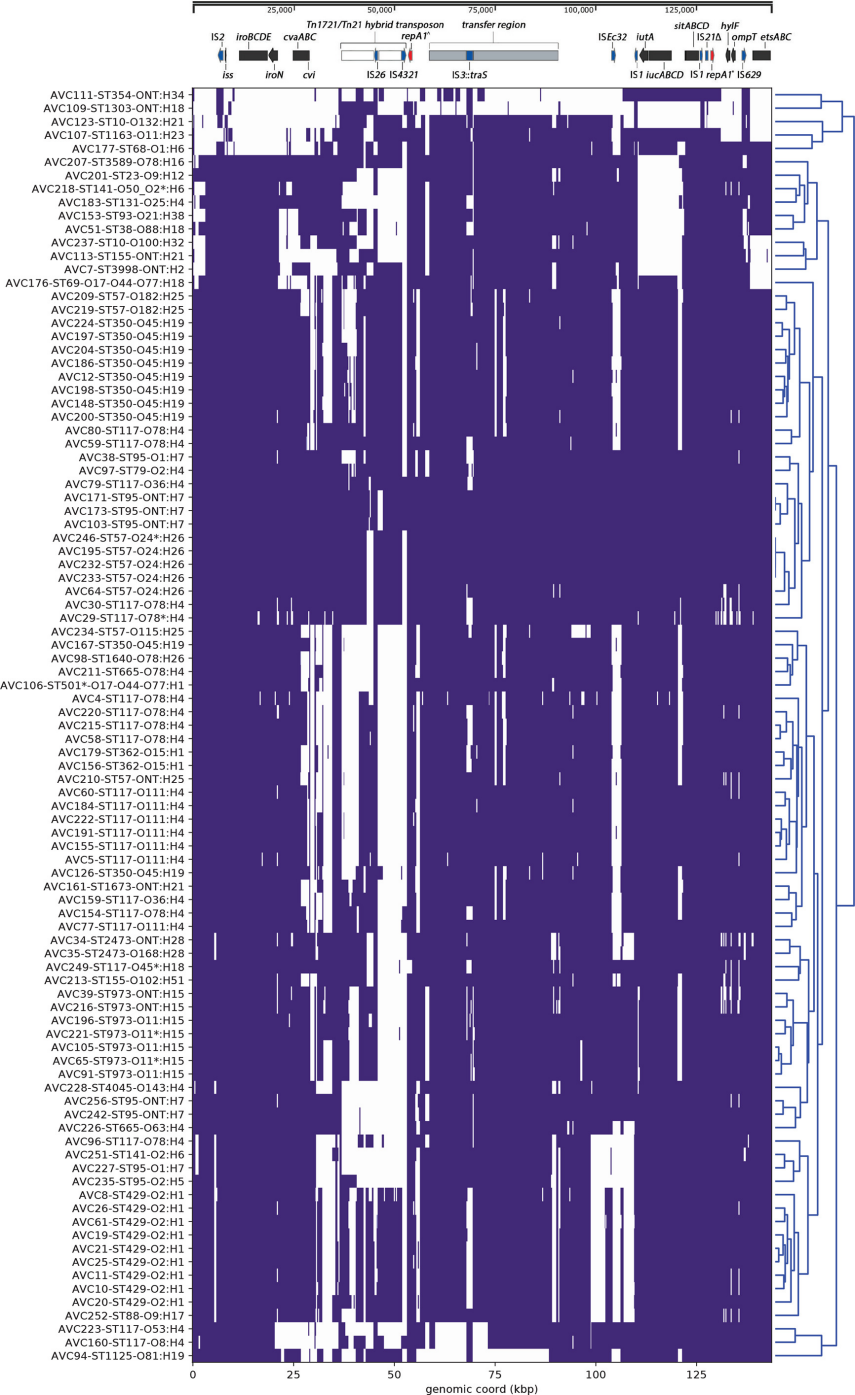
Mapping of short-reads indicating the presence of ColV-like virulence plasmids. Purple colour indicates a median depth of 10 or greater at a given 250 bp bin, whereas white space indicates the inverse. Clustering of rows on this heatmap is based on similarity between the coverage profiles of the isolates, while a schematic of pCERC4 is shown above the heatmap to provide an overview of the genetic elements that were present or absent based on this analysis. Key: *repA1*^, IncFII *repA1* gene; repA1*, IncFIB *repA1* gene.

A preliminary analysis of the frequency of virulence plasmid-associated VAGs in our collection suggested that ColV-like plasmids were a feature of these Australian APEC. To examine this further we used a recently published ColV plasmid sequence (KU578032) as a reference to map Illumina short-reads derived from our 95 APEC genome sequences. A bespoke python script was used to construct a visualization of the coverage of mapped APEC reads (Fig. 4). The utility of this approach is demonstrated by the observation that all 11 APEC isolates that did not carry *iutA* and *iucD*, as determined by ARIBA (e.g. isolates AVC51 and AVC207), lacked reads mapping to the corresponding region of the reference plasmid pCERC4, as shown in Fig. 4. Our analyses also showed that genomes belonging to the same ST and e-serotype shared high similarity in their read mapping profiles and therefore commonly clustered together, suggesting they may carry closely related plasmids. Along with the high carriage of virulence plasmid-associated VAGs and IncFIB/IncFII *repA* genes, our data suggest that ColV/ColBM-like plasmids are common in *intI1*-positive Australian APEC, an observation that mirrors studies elsewhere [104]. However, while Fig. 4 strongly suggests that the VAGs are within a plasmid, the analysis allows mapped reads to be recruited from any part of the genome and does not confirm the co-localization of the VAGs on a ColV-like plasmid backbone.

Notably, reads from samples AVC103, AVC171 and AVC173 all mapped extensively across the reference sequence pCERC4, which is sourced form a human commensal ST95 strain [105]. All such samples were also identified as ST95; this ST is well documented in associations with poultry meats, poultry disease and human extraintestinal infections [71]. Therefore, it is possible that this strain and/or plasmid may be closely related to those of the ST95 APEC samples under study. Long read sequencing of these samples and other Australian APEC would assist in the determination of VAG and AMR gene context and allow for comparative genomic investigations that may infer the movement of microbial populations and/or their plasmid content between different environmental contexts.

It is important to note two limitations of the study: sampling was inconsistent by state, and our collection is biased through selection based on carriage of *intI1*. Despite these limitations our study suggests: (i) that while *intI1*-positive Australian APEC are phylogenetically and serotypically diverse, particular lineages, such as CC117 and CC350, appear to constitute the primary health burden in the poultry sector; and (ii) these APEC carry large virulence plasmids which may also harbour genetic elements conferring resistance to antibiotics used to treat UTIs, such as trimethoprim and sulfamethoxazole, as well as genes encoding resistance to a wide array of first-generation antibiotics. Further work is required to investigate the genetic context of the AMR genes described here, and regions that abut insertion sequences such as IS*26*, because many AMR genes are mobilized by IS*26* [106, 107]. While many of the APEC isolates under investigation were determined to be genotypically MDR, none carried genes conferring resistances to critically important antibiotics such as colistin, extended-spectrum beta-lactams or fluoroquinolones, except one sample that carried SNPs linked with resistance to fluoroquinolones. A subset of APEC, such as ST117 and ST95, are phylogenetically and genotypically similar to *E. coli* that cause human extraintestinal infections, highlighting a potential zoonotic risk. Efforts are needed to ensure poultry are restricted in their capacity to be a reservoir of pathogenic *E. coli*, particularly those that may pose a zoonotic risk and carry broad-host conjugative plasmids containing VAGs and AMR genes.

## Data Bibliography

1. Zankari E, Hasman H, Cosentino S, Vestergaard M, Rasmussen S, et al. Identification of acquired antimicrobial resistance genes. J Antimicrob Chemother 67, 2640–2644 (2012).
2. Siguier P, Perochon J, Lestrade L, Mahillon J, Chandler M. ISfinder: the reference centre for bacterial insertion sequences. Nucleic Acids Res 34, D32–D36 (2006).
3. Inouye M, Dashnow H, Raven LA, Schultz MB, Pope BJ, Tomita T, Zobel J, Holt KE. SRST2: Rapid genomic surveillance for public health and hospital microbiology labs. Genome medicine 6:(11), 90 (2014)
4. Joensen KG, Scheutz F, Lund O, Hasman H, Kaas RS, et al. Real-time whole-genome sequencing for routine typing, surveillance, and outbreak detection of verotoxigenic *Escherichia coli*. J Clin Microbiol 52, 1501–1510 (2014).
5. Carattoli A, Zankari E, Garcia-Fernandez A, Voldby Larsen M, Lund O, et al. In silico detection and typing of plasmids using PlasmidFinder and plasmid multilocus sequence typing. Antimicrob Agents Chemother 58, 3895–3903 (2014).
